# First Steps towards Underdominant Genetic Transformation of Insect Populations

**DOI:** 10.1371/journal.pone.0097557

**Published:** 2014-05-20

**Authors:** R. Guy Reeves, Jarosław Bryk, Philipp M. Altrock, Jai A. Denton, Floyd A. Reed

**Affiliations:** 1 Department of Evolutionary Genetics, Max Planck Institute for Evolutionary Biology, Plön, Germany; 2 Department of Evolutionary Theory, Max Planck Institute for Evolutionary Biology, Plön, Germany; University of Missouri, United States of America

## Abstract

The idea of introducing genetic modifications into wild populations of insects to stop them from spreading diseases is more than 40 years old. Synthetic disease refractory genes have been successfully generated for mosquito vectors of dengue fever and human malaria. Equally important is the development of population transformation systems to drive and maintain disease refractory genes at high frequency in populations. We demonstrate an underdominant population transformation system in *Drosophila melanogaster* that has the property of being both spatially self-limiting and reversible to the original genetic state. Both population transformation and its reversal can be largely achieved within as few as 5 generations. The described genetic construct {*Ud*} is composed of two genes; (1) a *UAS-RpL14.dsRNA* targeting RNAi to a haploinsufficient gene *RpL14* and (2) an RNAi insensitive *RpL14* rescue. In this proof-of-principle system the *UAS-RpL14.dsRNA* knock-down gene is placed under the control of an *Actin5c-GAL4* driver located on a different chromosome to the {*Ud*} insert. This configuration would not be effective in wild populations without incorporating the *Actin5c-GAL4* driver as part of the {*Ud*} construct (or replacing the UAS promoter with an appropriate direct promoter). It is however anticipated that the approach that underlies this underdominant system could potentially be applied to a number of species.

## Introduction

Genetic underdominance at a given locus occurs when heterozygotes have a lower fitness than both homozygotes; *i.e.* heterozygote disadvantage [1], [2]. The mechanism of population transformation can be illustrated for an underdominant transgenic construct ({*Ud*}). If it is assumed that both wildtype (+/+) and transgenic homozygotes ({*Ud*}/{*Ud*}) have equal fitness (though this is not a necessary requirement), and if the frequency of the {*Ud*} allele is raised above a 0.5 threshold frequency by releases of {*Ud*}/{*Ud*} homozygotes into a target wildtype population, then {*Ud*} alleles will likely eliminate wildtype alleles at this locus over the course of subsequent generations. The elimination of wildtype alleles can occur even after releases have ceased [1], [3]–[5]. This mechanism is a consequence of the Hardy-Weinberg principle. The Hardy-Weinber principle states that the rarer wildtype alleles (+) spend proportionally more time as low fitness heterozygotes than the {*Ud*} allele [6]. This population transformation process is inherently reversible. as wildtype homozygotes could also be released to traverse the population frequency threshold in the opposite direction. Consequently, underdominant population transformation can be thought of as an evolutionary bi-stable switch. The switch works such that exceeding a threshold frequency dictates which allele will ultimately be fixed. Crucially, the transgenic homozygote can be less fit than the wildtype homozygotes and still attain stable fixation. This results in the three well-known properties of underdominant population transformation [3]–[5], [7]–[9]: (1) geographically self-limiting to specific populations, (2) reversibility to original wild state, and (3) linkage to deleterious transgenes does not prevent population transformation. It is important to note that the driving effect of {*Ud*} will be centered on its insertion site with the remainder of the genome being free to recombine with wildtype chromosomes.

The implementation of the {Ud} system described here relies on a knock-down and rescue system in a transgenic construct. Our transgenic construct targets an endogenous haploinsufficient gene (with expression control of the knock-down gene divided between two loci using the GAL4/UAS system, [10], [11]). Haploinsufficient genes are ones where null (amorphic) or hypomorphic mutations cause a dominant phenotype. A well-studied class of haploinsufficient genes are the *D. melanogaster* Minute loci (reviewed in [12]). Our approach relies on dominant RNAi-based suppression of an endogenous (present in the wildtype genome) haploinsufficient gene. The RNAi mediated knock-down of the endogenous gene is rescued by an RNAi-insensitive copy of the gene also in the same *{Ud}* construct. Expression from a single rescue allele in a *{Ud}/+* heterozygote is insufficient to completely rescue the knock-down. The allele thereby produces a fitness reducing haploinsufficient phenotype. Two alleles in a transgenic homozygote *{Ud}*/*{Ud}* effectively rescue the haploinsufficient phenotype (see strategy overview in Figure S1 in File S1). Note that our heterozygote is technically a hemizygote, but we are using the heterozygote designation for convenience in keeping with earlier underdominance literature. The endogenous haploinsufficient gene targeted in the construct described here is *RpL14* (located on chromosome arm 3 L with a cytogenetic location of 66D8), which is a cytoplasmic ribosomal protein (CRP). Heterozygous amorph or hypomorph mutations in *RpL14* result in a classic strong Minute phenotype with delayed development, slender scutellar bristles and reduced female fertility [13]. It is noteworthy that haploinsufficient mutations in the 88 CRP genes of *D. melanogaster* very likely correspond to 64 of the 65 known Minute loci [12]. Haploinsufficiency of CRP genes has been reported in a wide range of organisms including yeast, Arabidopsis, Drosophila, zebrafish, humans and mice (see references in [12], [14]).

Herein we report a transgenic construct {Ud}, a simple modification that, when introduced into target populations of insects at a sufficiently high frequency through mass release of individuals, could be used to push disease refractory genes into wild populations.

## Materials and Methods

### Development of UAS-dsRNAi.RpL14 Knock-down of RpL14^[+]^

The *RpL14* gene (CG6253, FBgn0017579) was selected as a representative CRP gene, which had previously been described as exhibiting a strong to extreme Minute phenotype [13]. To identify an approximately 70 bp region of *RpL14* to target by RNAi knock-down we sought to fullfill the following four criteria in the wildtype exon sequence; (1) No predicted off-target effects (default setting with ‘Off-Target Size’ set to 16 bp, http://www.flyrnai.org/cgi-bin/RNAi_find_primers.pl), (2) No regions with a high number of non-degenerate codons ATG or TGG, (3) target must be present in all alternative splicing variants, and (4) the region exhibits a high degree of structural accessibility (according to default settings of S-Fold, http://sfold.wadsworth.org/cgi-bin/sirna.pl). When these criteria were applied it was not possible to target a single contiguous region. Hence two non-contiguous regions were selected (called block A and B). Both regions happened to be on exon 2. The sequence of *RpL14.dsRNA* is shown in Figure S2 in File S1. An inverted repeat of the targeting sequence was assembled and cloned into the pUASattB plasmid (Genbank: EF362409) at the multiple cloning site, downstream of the UAS promoter, Figure S3 in File S1. SURE 2 supercompetent *E. coli* cells (Agilent Technologies) were used for bacterial transformation to ensure the stability of the short hairpin structure. The toxicity of *RpL14.dsRNA* in targeting *RpL14^[+]^* was experimentally demonstrated using flies transformed with *{ attR, w^[+mC]^, UAS-RpL14.dsRNA}51C or 51D* crossed to an *Actin5c-GAL4* driver [15] *P{w[+mC] = Act5C-GAL4}25FO1*). As expected [16], this resulted in 100% lethality of individuals hemizygous for both constructs (data not shown).

**Figure 1:**
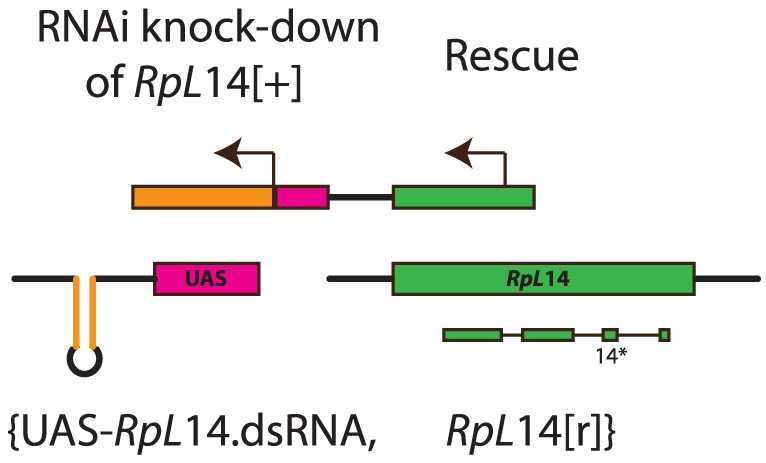
Configuration of the proof of principle underdominant transgenic construct {*Ud*}. The first gene of the construct encodes a dsRNA RNAi knock-down (orange) under the control of a UAS promoter 10 (magenta). The dsRNA targets RNAi to 72 bp of exon 2 of the endogenous wildtype gene *RpL*14^[+]^. The second gene (green) of the construct is a rescue gene *RpL*14^[r]^ that is insensitive to the RNAi knock-down (see Figure S1 in File S1 for strategy overview). *RpL*14^[r]^ is a complete *RpL*14 gene, including all its original regulatory regions, into which 14 synonymous mutations (14*) have been strategically introduced in exon 2 (Figure S3 in File S1). Expression of the RNAi knock-down is under the control of an *Act*5*c*-GAL4 driver at a remote locus.

The *RpL14.dsRNA* gene was placed under the control of a UAS promoter (Figure 1) to facilitate testing the promoters used for driving the RNAi knock-down expression. The targeted gene had the added benefit that the system would quickly be decoupled in the offspring of any accidentally escaped flies (see also discussion).

### Development of rescue RpL14^[r]^

The *RpL14* region was amplified with Phusion Taq (Finnzyme) using DNA prepared from the genome reference stock (Bloomington Drosophila Stock Center No. 2057: *y* *^[1]^* *; Gr22b* *^[1]^* *Gr22d* *^[1]^* *cn* *^[1]^* *CG33964^[R4.2]^ bw* *^[1]^* *sp* *^[1]^* *; LysC* *^[1]^* *MstProx* *^[1]^* *GstD5* *^[1]^* *Rh6* *^[1]^*). This was done using the following primers NotI -5′TATGCGGCCGCttgattagtttcctggccactt and EcoRI-5′TATGAATTCaaggcataagagctttgaatcg. This resulted in amplification of 3 L: 8593592..8596494. To help ensure that all unidentified regulatory regions of *RpL14* were incorporated, fragments of the flanking genes were also included in the PCR product. The endogenous *RpL14* fragment was cloned into the HindIII site immediately upstream of the UAS promoter in the pUASattB plasmids already containing the UAS- *RpL14*.*dsRNA* sequence (see above), Figure 1. It may be noteworthy that two intronic snoRNAs were also cloned as part of the fragment, though only exonic sequences are targeted by the *dsRNAi.RpL14* gene.

The 14 synonymous mutations that conferred insensitivity of *RpL14^[r]^* to RNAi targeting were introduced into exon 2 by synthesizing a new sequence (DNA synthesis done by DNA2.0, Menlo Park, USA). This was then ligated into a plasmid in the place of the corresponding wildtype sequence, using standard cloning techniques. As far as possible, the introduced mutations preserved the same balance between *D. melanogaster* preferred and unpreferred codons. The insensitivity of *RpL14^[r]^* to RNAi targeting and its effectiveness as a phenotypic recuse gene was demonstrated by its ability to rescue the otherwise 100% lethality of *UAS-RpL14.dsRNA* in the presence of Act5C-GAL4 expression (see Figure S4b in File S1).

### Generation of outbred transgenic stocks

Germline transformants were generated by inserting the *{Ud}* plasmid (Figure 1) into the 86Fb (3R:7634329, [17]) landing site (injections done by BestGene, Chino Hills, USA). Heterozygous transformants *y* *[1]* w[*]; *M{3x-P3-RFP, {attR, w^[+mC]^, RpL14^[r]^, UAS-RpL14.dsRNA, attL}}86Fb/+* were outcrossed for >3 generations to a mixed assortment of the following wild derived stocks, the wildtype lines are Bloomington Drosophila Stock Center No. 3848, CO 3 (NY, USA); Bloomington No. 3885, Wild 5A (GA, USA); Aquadro lab (Cornell University), B96 (Bejing, China), and 9 stocks acquired from the wild in 2009 in Kiel and Plön, Germany. The crossing scheme outlined in Figure S5 in File S1 details subsequent steps in the generation of the following two stocks that were used in varying frequencies to initiate all population experiments: {Act5C-GAL4}/CyO; *{Ud}*86/*{Ud}*86 and {Act5C-GAL4}/CyO: +/+. As the genotypes differ only on the third chromosomes, only the third chromosomes genotype is generally used in the main text.

Note that while all chromosomes were outcrossed for >3 generations, the maintenance of the second chromosome balancer CyO in the experiments keeps the original outbred second chromosomes in a permanently heterozygous state preventing the large fitness cost (>90%, [18]) associated with chromosomal isogenicity. This will also have the effect of preventing recombination during the population experiments on the second chromosome, which will not be the case for the first and third chromosomes.

**Figure 2:**
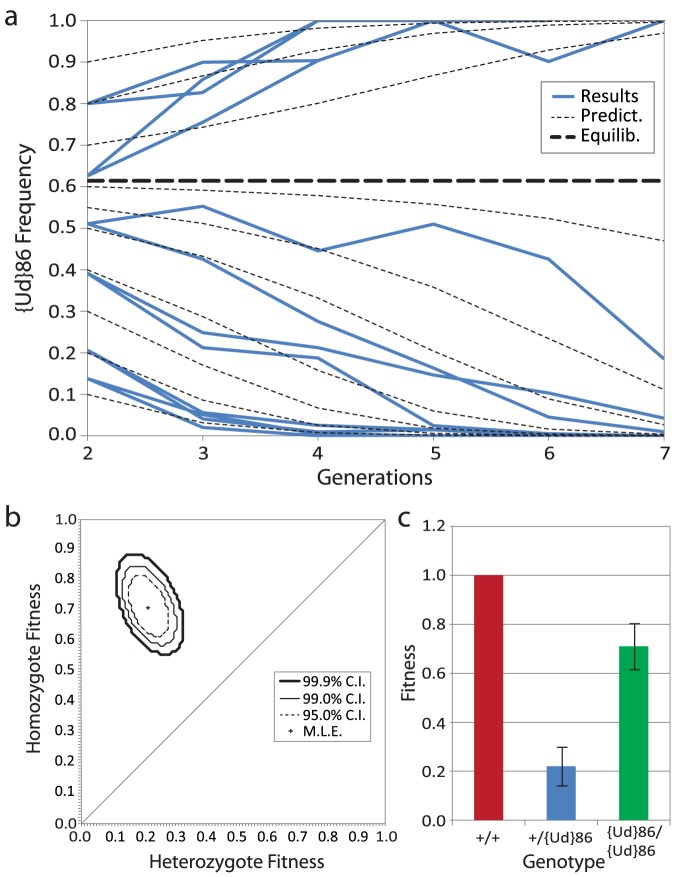
{*Ud*}86 population experiments demonstrating strong underdominance. (**A**) Multigenerational population experiments showing the frequency change in transgenic individuals from various starting frequencies (blue lines). Populations with starting values higher than the estimated threshold allele frequency of 0.61 (straight bold dashed line) proceed to fixation while those below result in loss of {*Ud*}86 (verified by crosses and PCR in all 12 populations). Dashed lines indicate predicted trajectories, under the maximum likelihood estimate (MLE) of fitness parameters shown in parts b and c. (**B**) Likelihood surface of relative fitness estimates. Fitness is inferred from the change in allele frequencies in A (**C**) MLE fitness estimates of the transgenic genotypes.

The stocks used to establish the experimental outbred populations were sequenced to detect any variation in the RNAi targeted region (3L:8594414..8594515). Using PCR primers 5′- TGGTCACCAACACAAGCAAC and 5′ GCCATGAAGGGAGGAGTACA. Sequenced stocks included Bloomington stock 3848,CO 3 (NY, USA); Bloomington stock 3885, Wild 5A (GA, USA); Aquadro lab, Bj96 (Bejing, China). Seven of the 9 stocks acquired from the wild in 2009 in Kiel and Plön (Germany) were also sequenced (2 stocks had died prior to sequencing). No polymorphism was detected. Sequencing also detected no polymorphism in 5 stocks resulting from the endpoints of the experiments shown in Figure 2. These stocks had been maintained as bottle populations for more than a year after the end of the experiment. DNA was prepared from pools of 10 individuals for each of the 5 stocks, PCR products were generated and cloned. A total of 20 RpL14 66D8 derived clones were sequenced and found to be invariant. The complete lack of polymorphism detected is consistent with the levels of variation observed in this region in large scale sequencing projects. For example based on 156 individuals with complete sequence data for this region there is just one polymorphic site (sampled in just two individuals [19]).

### Epi-fluorescent genotyping of flies

Genotyping of flies was done by surveying for the RFP fluorescence of the *3x-P3-RFP* gene at the {*Ud*}*86* landing site. Scoring of fluorescence was done on a Leica MZ10 F epi-fluorescent microscope with a Leica EL 6000 light source in a darkened room. Flies were lightly anesthetized using CO_2_. As all flies had pigmented eyes, RFP scoring in the eye was unreliable due to quenching, however the ocelli could reliably be used. For RFP scoring a Leica-dsRED filter set was used (excitation 545/30 nm, emission 620/60 nm). For *GFP* expression scoring a Leica-GFP2 filter set was used (excitation 480/40 nm, emission 510 nm).

### Fly rearing

All flies were maintained on standard media (http://flystocks.bio.indiana.edu/Fly_Work/media-recipes/bloomfood.htm) in an incubator at 24°C under a 14:10 hour light/dark cycle. Either 50 mL vials or 300 mL bottles, as specified below, containing food were used. When flies were added to a new container, yeast was added to the food surface to stimulate egg laying. When adults were cleared from a bottle two kimwipes were added to reduce emerging adult death rates (from becoming stuck in liquefied food).

### Population experiments

The experiment to measure allele frequency change over multiple generations was initiated with 20 mated females from the above two outbred homozygous stocks, at either 20%, 50% or 80% initial *{Act5C-GAL4}/CyO; {Ud}86/{Ud}86* frequencies (*{Ud}*86 homozygotes). The remainder of individuals were *{Act5C-GAL4}/CyO: +/+* (wildtypes). These already mated females were allowed to lay eggs in vials for three days and then cleared. These homozygous female parental flies were termed the G0 generation. The purpose of this generation was to minimize the possibility that differences in the condition or maturity between stocks did not strongly bias starting frequencies. The next generation, G1 was genotypically identical to generation G0, but larval development conditions are better controlled. Up to 100 G1 adults from a vial were collected, scored for sex and RFP using epi-fluorescence microscopy, and then allowed to lay eggs in a new bottle for 3 days before being cleared. Following 12 additional days of larval development, up to (approximately) 100 G2 offspring were collected scored for sex and RFP and introduced into a bottle for three days. This sequence was repeated for all subsequent generations. The G2 generation is the first in which heterozygotes are present (Figure 2a) and consequently it is the first generation in which an underdominant effect could be detected. This is why generation 2 is the first generation shown in Figure 2a.

An egg laying time of 3 days and generation spacing of 15 days, from the time of initial addition of flies to each bottle, was chosen to maximize population sizes and minimize possible effects of developmental delay, *i.e*. the balance between early eclosing genotypes dying in the food and thus underscored and later eclosing genotypes being underscored. Using the curve of development time, Figure S4a in File S1, the observation that eggs were consistently observed to have been laid on all three days, and assuming 100% homozygous genotype survival, this would only result in a relative loss of 7% of the expected heterozygous genotypes. This is not sufficient to explain the 78% fitness reduction estimated for heterozygotes, which as discussed in the main text is likely to result from a combination of pleiotropic effects of reduced CRP expression of in some tissues (see also Figure S1 in File S1).

### Population experiment statistical tests

The strategy of scoring a dominant RFP marker to infer the frequency of {*Ud*}*86* makes minimal assumptions regarding fitness. The frequency of {*Ud*}*86* was estimated as *p* = 1−√(*R* ^−^/*n*), where *R* ^−^ is the fraction of non-RFP expressing adults and *n* is the total number scored (plotted in Figure 2a; data is given in Table S1 in File S1). A likelihood-ratio test, related to but having better properties than the standard χ^2^ test [20], was used to analyze the counts of RFP and non-RFP males and females between generations and search for maximum-likelihood parameter value estimates over a grid of transgenic homozygote and heterozygote fitness values (relative to a wildtype fitness set to one). This was done by minimizing the G-statistic of a likelihood-ratio G-test [21], [22] for predicted changes in frequency between generations (*cf*. [23]) according to the fitness of each genotype and their corresponding frequency in the population in non-overlapping, random-mating Wright-Fisher generations [24]. According to standard deterministic Wright-Fisher model assumptions the expected allele frequency in the next generation *p*′, is given by the allele frequency in the current generation *p*, the homozygote and heterozygote fitness values *w*_+/{Ud}_ and *w*_{Ud}/{Ud}_, and the average fitness in the population, 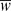. Change in allele frequency then follows 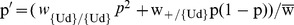



For a given set of fitness values this generates an expected value that the observed data can be compared to in a likelihood framework [25]. Assuming independence, both in allele frequency changes across replicates and between sets of generations, allows the sum of the log-likelihood ratios of individual frequency changes to be used to generate a joint/composite likelihood surface over the full data set (equivalent to multiplying probabilities using the independence rule). According to Wilks' theorem, and the assumption that the errors are normally distributed according to the central limit theorem of many small effects, the test-statistic is expected to be asymptotically χ^2^ distributed [26], which was used to generate joint confidence intervals (plotted in Figure 2b). Profile likelihoods derived from the joint surface were used to generate the confidence intervals for individual parameter estimates (plotted as error bars in Figure 2c).

### Confirming homozygosity of stocks

Homozygosity was confirmed by crossing large numbers of (*n*>30) tester males to virgin females (from a *w[1118]*/, *Dp(2;Y)G*, *P*{*w[+mC] = hs-hid*}Y virginizer stock) and determining whether all offspring were RFP fluorescent or all non-fluorescent. In addition the following primers were used on single fly DNA extractions 5′ gggccaaagtgtaaataactgg and 5′ aaaatgtccattactttggtgct to give a 136 bp PCR product (3R:7634237..7634372) identifying the presence of wildtype third chromosomes (*+*) and a theoretical product of >10 kb for *{Ud}86*. Presence of {*Ud*}*86* could be confirmed by using 5′ actttccttccgatggacct and 5′ aatgaccaccgtctttcagc resulting in a 135 bp PCR product from the RFP gene.

### Development time and non-mendelian distortion

In order to more precisely quantify development time and egg-to-adult departure from expected Mendelian ratios among the three genotypes, a stock was generated with GFP expression from the 86Fb cytological insert site as a proxy for wildtype. This GFP stock had the genotype w[*];CyO/P{w[+mC] = Act5C-GAL4}25FO1; M{*3x-P3-GFP*}*86Fb* and was generated from a modification of a plasmid (pGFP-lox-attB_12.gb.1) kindly given to us by Dr. Johannes Bischof (University of Zurich).

Vials were used in this experiment and crosses were set up according to the parental types indicated in Figure S4b in File S1. The parental flies were transferred to new vials each day, for a total of 10 days, and the resulting newly eclosing offspring were scored, for sex and RFP/GFP presence/absence, each day over the following 25 days. The data is given in Table S2 in File S1.

The heterozygous to homozygous parental back-crosses allowed the rate of erroneous genotype scoring to be estimated. Vials for each cross were given an alphanumeric identifier and offspring were scored “blind” each day without knowledge of the parental cross. A total of 9 offspring with genotypes not allowed by the cross (e.g. RFP/RFP homozygous offspring from GFP/RFP x GFP/GFP parents) were detected out of 2,636 offspring from crosses where these types of errors could be detected. Assuming that half of the erroneous genotype scores are detectable (the remaining falling into an allowed genotype category for a particular cross), the predicted genotyping error rate is 0.68%.

### Dry weight assay and statistical tests

Five sets of 10 one-day old adult flies of each sex and genotype were frozen overnight then dried in a heater for six hours at 30°C. The flies were weighed in batches of 10 at a time in order to get more accurate measurements. This data is given in Table S3 in File S1 and was tested using a standard ANOVA and F-distribution [27].

### Quantitative TaqMan PCR

The total amount of *RpL14* mRNA and the ratios of endogenous wild-type *RpL14* transcript and the rescue construct were assessed with a two-step quantitative reverse-transcription PCR (RT-qPCR). RNA was isolated from 10 adult flies for each sex or 10 unsexed larvae using Trizol (Invitrogen) and DNase treated using a PureLink RNA Mini Kit (Invitrogen). RNA quality was assessed using a NanoDrop spectrophotometer and 100 ng was used for cDNA synthesis using a Maxima First Strand cDNA synthesis kit for RT-qPCR (Fermentas). 1 µL of the resulting reaction was used as a template for qPCR, using TaqMan Fast Master Mix (Applied Biosystems) for determining the relative ratios of wild-type and rescue transcripts or SYBR Green Fast Master Mix (Applied Biosystems) for total *RpL14* mRNA levels. Each sample was run in triplicate on an ABI 7900HT machine (Applied Biosystems). Both wild-type and rescue PCR products were amplified with the same primer pair spanning an intron-exon boundary; 5′ TCTTTCCGGTTAGCGTCAT; 5′ CGCCAGTCAGAGGACCAT. Expression of wild-type and rescue transcripts were detected with TaqMan MGB probes (Applied Biosystems) labeled with FAM (wild-type) and VIC (rescue). Both probes had the same base composition, but differed by two bases, allowing for highly specific detection of wild-type and rescue transcripts and facilitating calculation of ratios of expression; probe-wild-type FAM-TTGCCAAGGCCTCCGC-MGB; probe-rescue VIC-TCGCCAAAGCCTCCGC-MGB. The specificity of the probes is demonstrated in Figure S6 in File S1. For normalization of the qPCR signal, we used the GeNorm method [28] and selected 8 genes previously suggested [29] as a good set for normalization of qPCR reactions. We tested these genes for stability of expression in our experimental conditions (in larvae and adults for all genotypes) across 6-log serial dilutions of cDNA template and identified three of these genes, FBgn0032882 (CG9320), FBgn0039259 (CG11781) and FBgn0002021 (CG10655) as the least variable in the flies and used these as normalization genes for the calculation of the normalization factor. *RpL14* mRNA levels are presented relative to the normalized geometric mean of the three normalization genes.

### Data deposition

The sequence and *{Ud}* plasmid is available from addgene.org with the ID 53220 and name {Ud}RpL14.Dm.

## Results

### The {Ud} construct

This system achieves underdominance through a “knock-down/rescue” system contained in the construct {*Ud*} (Figure 1). {*Ud*} employs dsRNAi knockdown (*RpL14.dsRNA*) of an endogenous CRP, *RpL14*, driven by the UAS promoter [10]. This knock-down gene encodes a dsRNA inverted repeat, targeting RNAi to 72 base pairs (bp) of the endogenous *RpL14* wildtype exons (*RpL14^[+]^*), no intronic bases are targeted by the dsRNA inverted repeat. The construct also contains a rescue (*RpL14^[r]^*) that is a complete copy of the wildtype *RpL14* gene (including its promoter and flanking regions, to maximize the likelihood that rescue is complete) where 14 synonymous mutations had been introduced within the 72 bp region targeted for RNAi by *RpL14.dsRNA* (Figures S2 and S3). The number and position of synonymous changes ensures that all ∼21 bp siRNA fragments potentially produced by Dicer proteins for RNAi targeting incorporated a minimum of 3 mismatches relative to the rescue gene *RpL14^[r]^*, intended to render it substantially insensitive to RNAi targeting.

In this proof-of-principle system of underdominance, the GAL4/UAS system [10] has been used to control expression of *RpL14.dsRNA*. This was done to permit flexibility in testing the promoters used for driving the RNAi knock-down expression and has the added benefit that the system would quickly be decoupled in the offspring of any accidental escaped flies. However, as the GAL4 driver is not part of the {Ud} construct in this example implementation the system is split across two loci and therefore *RpL14.dsRNA* is only expressed in a GAL4 background.

### {Ud} Genomic integration & stain development

{Ud} was integrated at an RFP marked attP/C31 landing site on chromosome three ([17]; cytogenetic location 86Fb), resulting in the genotype *M{3x-P3-RFP, {attR, w^[+mC]^, RpL14^[r]^, UAS-RpL14.dsRNA, attL}}86Fb*, hereafter referred to as *{Ud}86*. To drive strong expression of the UAS-RNAi knock-down in at least some tissues, which can potentially experience haploinsufficiency, a constitutive second chromosome *Actin5c-GAL4* driver was selected [15] *P{w[+mC] = Act5C-GAL4}25FO1*.

### Phenotype of {Ud}86

Given the strong fitness reduction in heterozygotes and the pleiotropic impact of CRP hypomorphs [12], [13], we examined life history and morphological traits that could correlate with this genotype. Two red-eyed phenotype stocks were generated for these experiments: *{Act5C-GAL4}/CyO; {Ud}86/{Ud}86* and; *{Act5C-GAL4}/CyO: {GFP}86/{GFP}86*. We unambiguously scored all three genotypes in each generation using the fluorescence of the 3x-P3-RFP marker, which is part of the landing site used in *{Ud}86*, and the fluorescence of *GFP* inserted into the same position to mark the alternative chromosome as a proxy for wildtype. We observed no morphological abnormalities in either transgenic genotype. Interestingly, heterozygotes do not exhibit the short and thin scutellar bristles that are a characteristic feature of the Minute phenotype and most *D. melanogaster* CRP mutations. Furthermore, no difference in dry weight was observed between adults of the three genotypes (males, *F* = 0.55, *P* = 0.591; females, *F* = 1.34, *P* = 0.298, Table S3 in File S1). However, in common with Minute phenotypes, heterozygotes exhibit a development time prolonged by approximately 20 hours (Figure S4a in File S1, *P*<1×10^−30^), while homozygotes {*Ud*}86/{*Ud*}86 exhibit no significant differences from wildtype homozygotes (Figure S4a and Table S2 in File S1). Also, the relative egg–to–adult viability of the heterozygous genotype was 20% to 50% lower than in homozygotes (Figure S4 and Table S2 in File S1).

### Population transformation using {Ud}

To more directly test genotype fitness over the entire life-cycle, without possible fitness bias effects due to expression of a fluorescent marker in the wildtype stock, an unmarked red-eyed stock containing a balanced *GAL*4 second chromosome was generated for subsequent experiments (Figure S5 in File S1): *{Act5C-GAL4}/CyO: +/+*. Note that if there is a fitness effect of RFP expression on our {*Ud*}{*Ud*} homozygote fitness estimate relative to wildtype is likely to be conservative. This wildtype-proxy stock is genotypically equivalent to our functional {*Ud*}86 stock (*{Act5C-GAL4}/CyO: {Ud}86/{Ud}86*) in every way, including the balanced GAL4 driver on the second chromosome, except for the absence of the {*Ud*}86 insert. To ensure a robust test of underdominance, both stocks were initially out-crossed for 3 generations to a mixed stock composed of globally derived lines. This reduced the probability of large fitness losses in “wildtype” individuals due to isogenisity [18], which might artificially inflate the relative fitness of the {*Ud*}{*Ud*} homozygote (note that the outbred second chromosomes are maintained in a permanently balanced state preventing recombination on this chromosome, see materials and methods for additional discussion).

To look for the frequency dependent fixation of alleles that is diagnostic of underdominance, replicated population bottles over a range of frequencies of {*Ud*}86 and + were initiated (Figure 2a and Table S1 in File S1). We scored {*Ud*}86 allele presence in each generation using the dominant fluorescence of the *3x-P3-RFP* marker. Figure 2a shows a consistent and rapid rise in the frequency of *{Ud}86* when above a threshold frequency estimated at *p* = 0.61 and a corresponding decline when below it. The elimination of either *+* or *{Ud}86* based on initial frequencies demonstrates the inherent reversibility of underdominant population transformation and also underlines the spatially self-limiting nature of underdominant population transformation. Using a maximum-likelihood approach the relative fitness of the two genotypes relative to a “wildtype”-proxy fitness defined as one was estimated (Figure 2b and c) as *+/{Ud}86* = 0.22 (0.16–0.28, 95% C.I) for the heterozygote and *{Ud}86*/*{Ud}86* = 0.71 (0.62–0.81, 95% C.I) for the homozygote. These fitness values represent a strongly underdominant system. If the *{Ud}86* allele is pushed above 61% in a population by transgenic releases, it is predicted to proceed to fixation.

Note that the observed egg-to-adult viability reduction alone is insufficient to fully explain the 70–80% reduction in fitness over the entire lifecycle relative to homozygotes (Figure 2b). Given the fundamental importance of protein synthesis to most metabolic processes we speculate that it is likely that the unattributed fitness reduction is the consequence of a large number of small pleiotropic effects. This is consistent with the multiple but generally subtle phenotypes reported for most CRP hypomorphs in *Drosophila* [12], [30], [31].

### Confirming RpL14^[r]^ expression and RpL14^[+]^ knock-down

**Figure 3:**
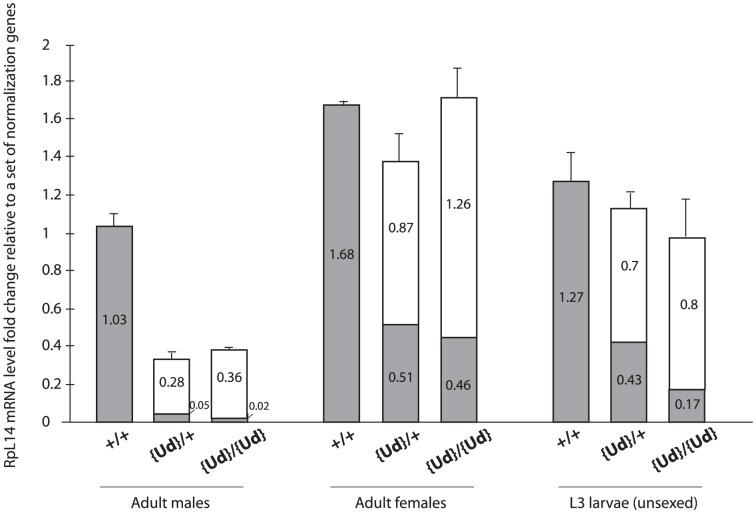
Genotypic levels of total *RpL*14 mRNA expression in groups of whole individuals. Amount of *RpL*14 mRNA in adults and larvae relative to three normalization genes. Height of bars indicate total amount of *RpL*14 mRNA based on SYBR green-based quantitative reverse-transcription PCR and each bar is split to represent the proportion of total *RpL*14 expressed from the *RpL*14^[r]^ gene in {*Ud*}86 (white) and the endogenous *RpL*14^[+]^ gene (gray), based on gene-specific TaqMan probes. Error bars represent 1 standard error for three biological replicates.

Estimates of total *RpL14* mRNA levels were performed using quantitative RT-PCR to establish that: (1) the RNAi knock-down was effective and substantially dominant; (2) that there was significant expression of the rescue. As anticipated, we observed a locus-specific RNAi induced reduction in wildtype *RpL14^[+]^* mRNA in both transgenic genotypes (Figure 3) and substantial expression of the rescue *RpL14^[r]^* (Figure 3 and S7). Given that *Act5C-GAL4* driven expression of a single *RpL14.dsRNA* gene in the absence of any *RpL14^[r]^* gene resulted in 100% lethality (data not shown, see also materials an methods) the generation of near wildtype numbers of viable offspring (Figure S4b in File S1) demonstrated that rescue expression was highly effective.

## Discussion

Currently, there are only three population transformation systems that have worked in laboratory experiments: a maternal poison-rescue system termed Medea [32], a homing-endonuclease base system termed HEG [33], and a different type of engineered underdominance based on reciprocal knock-down rescue termed UD^MEL^ [34]. Neither Medea nor HEG achieved complete fixation in experimental populations, with the stable equilibrium frequency of wildtype alleles remaining at approximately 0.1 for Medea (Figure 1F in [34]) and >0.1 for HEG (Figure S5 in [33]). Complete fixation was achieved for UD^MEL^ and {*Ud*}86 (Figure 2b). A potential concern is that persisting wildtype alleles could facilitate selection for resistance by the insect to the driving properties of HEG or Medea, or selection for resistance by the pathogen to the linked disease refractory gene [35]. While it is possible that future developments of these systems could address this issue [36] it is noteworthy that the underdominant approach demonstrated here reduces the probability of this complication by rapidly eliminating all wildtype alleles within a population (Figure 2a). However, while this does not entirely eliminate this concern; two additional areas warrant future experimental attention. The first is selection for resistance to {*Ud*}, e.g. by genomic duplication or up-regulation of the targeted haploinsufficient gene. The second, which is specific to the targeting of CRP genes, is the potential impact that high frequency viral infections could have in increasing the fitness of {*Ud*} heterozygotes. This possibility is raised by the finding that infections of the Drosophila C virus (DCV) are inhibited by knocking-down many CRP genes [37]. If future experiments validate either concern, both could potentially be ameliorated to a significant extent through: 1) selecting promoters for knock-down expression which maximize the fitness of transgenic homozygotes; 2) simultaneous targeting of more than one haploinsufficient gene in a single {*Ud*} construct, one of which is not a CRP gene (e.g. see Table 4 in [38]). The range of possible genotype configurations in the alternative engineered underdominance approach of UD^MEL^ allow for much lower population transformation thresholds, but at the cost of geographic stability and reversibility.

**Figure 4:**
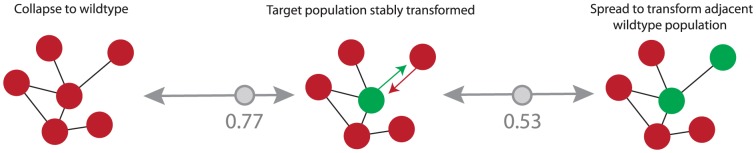
Geographic Stability. Interconnected populations are represented as circles, (red wildtype and green {*Ud*}86 transformed population). If a single target population is transformed (center) migration can result in two undesired transitions; collapse of population transformation (left), or spread (right). We assumed a migration rate of 0.065 every generation and the fitness configuration described in Figure 2b. Then collapse is only likely to occur if the allele frequency of {*Ud*}86 drops below *p* = 0.77. Spread is only likely to occur if {*Ud*}86 reaches *p*>0.53 frequency in an adjacent wildtype population.

The initiation of underdominant population transformation is undoubtedly a more resource intensive approach than for HEG [33] or Medea [32], where very small numbers of individuals can be released. For our {*Ud*}86 example this would require releases to exceed an allele frequency of 61% in a given wild population (Figure 2a). Consequently, an underdominant approach would probably be impractical if transformation of a large number of substantially isolated populations was required, or if populations were composed of multiple hybridizing sub-species (e.g. *Anopheles gambiae*). In such circumstances, the increased potential of *Medea* and HEG to readily spread between populations and subspecies would be much more efficient. However, where spatial control is valued, Figure 4 illustrates the extent of the geographic stability *{Ud}86* could exhibit even with substantial levels of migration [7] (see also File S1).

Obvious candidates for {*Ud*} transformation include the mosquitoes *Aedes aegypti* [39], *Culex quinquefasciatus* [40], and *Anopheles stephensi* [41], where refractory genes have been developed for dengue fever [42], avian malaria [43], and human malaria [44], [45].

Implementation of the UD^MEL^ approach in non-model pest species requires the identification and characterization of maternal and zygote expressed genes and promoters within the right range of expression and fitness effects. In contrast, the facts that *RpL*14 was the first gene selected for the development of this {*Ud*}86 technique, and Act5c the first driver of the RNAi expression chosen, suggest that our approach may be readily applied in other non-model organisms. The haploinsufficiency and the deleterious nature of CRP mutations is well conserved across wide evolutionary distance with examples in yeast, Arabidopsis, Drosophila, zebrafish, humans and mice (see references in [12], [14]). Consequently, CRPs should represent a rich source of genes that can potentially be targeted by {*Ud*} constructs in any sexually reproducing organism. While there has been no empirical demonstration of linking a disease refractory gene to a population transformation system, it is notable that due to the larger than 3 fold fitness reduction of *+/{Ud}86* heterozygotes, relative to *{Ud}86/{Ud}86* homozygotes (Figure 2b), it is expected that *{Ud}* would be well suited to fixing even strongly deleterious linked refractory genes [35]. Furthermore, it is anticipated that the spatial controllability and complete reversibility of {*Ud*} population transformation would prove a major advantage in attracting public and regulatory acceptance of this type of self-sustaining technology [1], [2], [32]–[34].

Further work remains to be done to more fully characterize this system. We suspect it is likely that the majority of the fitness reduction in heterozygotes is directly due to RpL14 haploinsufficiency (because this is a general propoerty of CRPs). However, we do not know what patterns of RNAi expession are sufficient in generating the reduction of RpL14 level. This can be investigated by substituting various GAL4 drivers into the *{Ud}*86 background by standard crosses. There may also be a fitness contribution from the knockdown of snoRNAs. snoRNAs are small nucleolar RNAs that play a role in mediating chemical modification of other RNA molecules [46] and are also expressed from the RpL14 transcript that exist in the transcript's introns. A number of CRPs described as haploinsufficient Minute loci also contain snoRNAs [47] and some of these snoRNAs are known to modify ribosomal RNA. Eventually, a more complete picture of what is driving CRP loci haploinsufficiency–via ribosomal interactions–might also include quantifying snoRNA abundance, and/or direct RNAi targeting of snoRNAs.

In the configuration reported here the RNAi knock-down gene was placed under GAL4/UAS control split across two chromosomes. This was done to permit the study of the system using different expression patterns and as a fail-safe for initial testing. However, the use of a two-locus GAL4/UAS system is not theoretically integral to our *{Ud}* approach. A GAL4 driver could be combined into the *{Ud*} construct, e.g., to generate *{RpL14^[r]^, UAS-RpL14.dsRNA, Act5C-GAL4}*. Alternatively the use of the GAL4/UAS system could be dispensed with entirely, e.g., by generating *{RpL14^[r]^,* Act5C -RpL14.dsRNA*}*. The complete elimination by replacement of the UAS promoter with the promoter used in a GAL4 driver is unlikely to result in a qualitative change in expression of the RNAi knock-down gene beyond a potential increase in homozygotes (due to having two alleles rather than one *RpL14.dsRNA* allele). Note that if it did prove problematic to recover homozygous stocks from initially heterozygous individuals (due to haploinsufficiency induced mortality) resulting from embryonic injections, it would be straightforward to inject a stock previously transformed with a co-dominantly marked ‘rescue only’ construct. This stock could then be segregated away in *{Ud}* homozygotes. However, given that heterozygotes are neither sterile nor inviable this is probably unnecessary for the insertion site examined (see Figure S4 in File S1).

The system presented here demonstrates a significant development in a population transformation system. The system has the potential to be portable not only in insects but across a wide range of sexually reproducing eukaryotic organisms. Furthermore, alternative dominant gene-knockout approaches to RNAi such as site–specific nucleases (CRISPRs, TALENs or ZFNs) could also potentially be utilized. However, before this can be used in truly wildtype populations the GAL4/UAS or equivalent system needs to be incorporated into a single loci or removed and replaced with an appropriate direct promoter.

## Supporting Information

File S1 Supplementary information file containing Figures S1–S7, Tables S1–S3 and supplementary equations.
(DOCX)
