# Supplementary material for: First Steps towards Underdominant Genetic Transformation of Insect Populations
Source: PLoS One. 2014 May 20;9(5):e97557. doi: 10.1371/journal.pone.0097557 (PMC4028297; doi:10.1371/journal.pone.0097557)
Supplement: File S1 — Supplementary information file containing Figures S1–S7, Tables S1–S3 and supplementary equations. (DOCX) [file pone.0097557.s001.docx]

**Figure S1. Strategy overview of how targeting a haploinsufficient gene in sensitive tissues could generate underdominance (heterozygote disadvantage).**


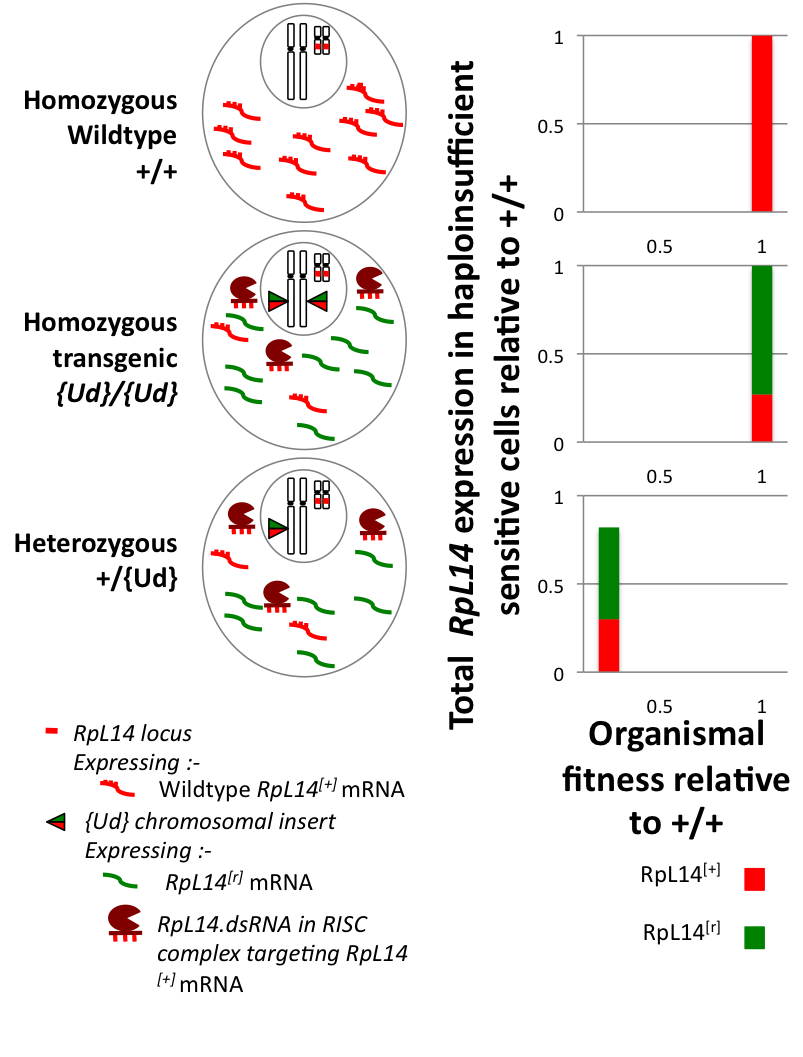


The haploinsufficient gene used to illustrate the *{Ud}* approach is *RpL14* which plays an essential role in protein synthesis as part of cytoplasmic ribosomes. A chromosomal integration of the *{Ud}* construct (Figure 1 and Figure S3) dominantly knocks-down wildtype *RpL14^[+]^* mRNA (through RNAi targeting by RISC complexes) which is rescued to a varying degree with *RpL14^[r]^* expression (which is insensitive to the RNAi). Expression from a single *RpL14^[r]^* allele in heterozygotes is insufficient to fully restore wildtype levels of total *RpL14* expression (y-axis) in at least some cells that are haploinsufficient sensitive, resulting in only a partial rescue of fitness (x-axis). While in transgenic homozygotes (possessing two rescue alleles *RpL14^[r]^*), expression in the haploinsufficient sensitive cells is sufficient to substantially or completely rescue wildtype fitness.

For underdominance to be generated it is only necessary that the knock-down (*RpL14.dsRNA*) is strongly expressed in at least some tissues that can experience haploinsufficiency. The use of any strong constitutive promoter is likely to satisfy this requirement. The first strong constitutive promoter tried during the development of the {Ud} approach, Actin5c, resulted in underdominance at two of the three attP/φC31 landing sites examined.

Assuming a strong and substantially dominant knock-down is achieved in at least some haploinsufficient cells, the primary determinant of a differential fitness between heterozygotes and homozygote transgenics is likely to be the expression of the rescue gene (*RpL14^[r]^*). Given that the rescue gene is always under the control of copies of its original regulatory sequences it is likely that positional effects of the genomic insertion site of {Ud} will have the greatest impact on rescue expression.

It is important to note that the graphs in Figure 3 and Figure S7 do not assay *RpL14* expression in exclusively haploinsufficient sensitive cells (as in the hypothecated graphs above), but do so for groups of whole individuals providing an average across all tissues. Given the limited nature of the described phenotypes of *Drosophila* Minute mutations it is likely that only a small subset of cells experience a haploinsufficient deficit in protein synthesis. Consequently, if the numbers of cells that are sensitive to haploinsufficiency is relatively small or the rescue locus does not replicate wildtype patterns of expression in all respects it is quite possible that assays of total *RpL14* expression averaged across all tissues may not exhibit detectable reductions in *RpL14* expression in heterozygotes (which is the case for L3 larvae, Figure 3). The main text describes the insertion of a *{Ud}* construct at cytogenetic location 86Fb of the *Drosophila melanogaster* genome, which is termed *{Ud}86*. However, the total levels of *RpL14* expression in populations of haploinsufficient cells remains to be determined and the organismal fitness of transgenic homozygotes is estimated as 0.71 and not 1.0 as illustrated above (see Figure 2). **Figure S2. Sequence of *RpL*14.dsRNA, its *RpL*14^[+]^ target and the corresponding sequence in the insensitive *RpL*14^[r]^ gene.**

The entire exon 2 of *RpL14* is shown. The first line of sequence shows the RNAi targeting *RpL14*.*dsRNA* (the sequence of the inverted repeat is not shown). Two non-contiguous blocks of sequences are targeted by *RpL14*.*dsRNA*. The linker between block A and B is positioned arbitrarily and has no similarity with any other sequence. Parallel lines show identity between the dsRNA and the *RpL14^[+]^* and *RpL14^[r]^* genes. While *RpL14^[+]^* is efficiently targeted for RNAi, the number and distribution of the 14 synonymous mutations in *RpL14^[r]^* prevents the same degree of knockdown by RNAi and rescues *RpL14* expression. The impact of changing synonymous nucleotides on codon bias of the 13 modified codons (*RpL14* is 166 codons in total) was minimised by selecting similarly preferred codons (see Materials and Methods). Block A is 41bp (targeting to 3L:8594414..8594454 of *RpL14^[+]^*) and block B 31bp long (targeting to 3L:8594485..8594515 of *RpL14^[+]^*). A short KpnI containing linker was inserted between the block A and B after checking that the linker did not cause any predicted off-target effects.

**Figure S3. Schematic of plasmid {*Ud*} used for fly injection.**

Diagram is a to scale schematic of the {*Ud*} plasmid injected into flies to place the construct at RFP marked landing sites using the φC31-based integration system. All labels are described in the text and Figure 1.

The sequence and *{Ud}* plasmid is available from addgene.org with the ID 53220 and name {Ud}RpL14.Dm.

**Figure S4. Egg to adult development time and survival.**

**(a)** The cumulative fraction of adults eclosing each day of development. Females are indicated with solid lines and males with dashed lines. The numbers in the legend give the average time to eclosion for each genotype in days. The difference between homozygotes and wildtype in development time is not significant (males Kolmogorov–Smirnov test *D*=0.0749, *n*_1_=674, *n*_2_=566, *P*=0.0634; females K-S test *D*=0.0445, *n*_1_=778, *n*_2_=715, *P*=0.452). The difference between pooled homozygotes and heterozygotes in development time is highly significant (males K–S test *D*=0.272, *n*_1_=1240, *n*_2_=966, *P*=2.56×10^-35^; females K-S test *D*=0.292, *n*_1_=1493, *n*_2_=1149, *P*=1.64×10^-48^).
**(b)** Relative abundance of offspring from various crosses. The columns correspond to the proportion of offspring genotypes from each type of cross. The labels along the bottom indicate parental genotypes. The proportion of homozygotes was set to 1. In all cases there is a deficiency of heterozygotes surviving to eclosion relative to homozygotes.

The horizontal bar indicates the expected relative proportion of heterozygotes. In all cases the heterozygous deficiency is significant (from left to right, d.f.=2, χ^2^=24.37, *P*=5×10^-6^; d.f.=1, χ^2^=8.33, *P*=0.0039; d.f.=1, χ^2^=11.34, *P*=0.000759; d.f.=1, χ^2^=4.40, *P*=0.0359; d.f.=1, χ^2^=37.68, *P*<10^-6^).

**Figure S5. Crossing scheme to generate outbred stocks for population experiments.**

Crosses were done reciprocally with as many individuals as possible (> 10) to maximize variation in the final stocks on all chromosomes (though all wild derived variation on the 2nd is effectively eliminated in the final crosses due to CyO balancing)

**Parallel cross 1**

Stock 4414 out crossed heterozygotes

*w[*]; T(2;3)ap[Xa], ap[Xa]/CyO; TM3, Sb[1]* x *w[*];+; M{3x-P3-RFP,w[[+mC],{Ud}}86 / +*

Select progeny: ap[+] w[+mC], RFP Cyo[-], Sb[1]

*w[*];CyO/+; TM3, Sb[1]/ M{3x-P3-RFP,w[+mC],{Ud}}86*

**Parallel cross 2**

Stock 2475 stock 4414

*y[1] w[*]; P{w[+mC]=Act5C-GAL4}25FO1/CyO, y[+]; +* x *w[*]; T(2;3)ap[Xa], ap[Xa]/CyO; TM3, Sb[1]*

F1 x

select progeny: ap[Xa], w[+mC], Cyo[+],Sb[1] (select against y[1] males)

*w[*]; T(2;3)ap[Xa], ap[Xa]/ P{w[+mC]=Act5C-GAL4}25FO1; +/ TM3, Sb[1]*

**cross 1 progeny cross 2 progeny**

*w[*];CyO/+; TM3, Sb[1]/ M{3x-P3-RFP,w[[+mC], {Ud}}86 x w[*]; T(2;3)ap[Xa], ap[Xa]/ P{w[+mC]=Act5C-GAL4}25FO1; +/ TM3, Sb[1]*

select progeny: CyO[-], RFP , Sb[1]

*w[*];CyO/ P{w[+mC]=Act5C-GAL4}25FO1; TM3, Sb[1]/ M{3x-P3-RFP,w[[+mC],{Ud}}86*

Bloomington stocks 4414, *y[1] w[*]; P{w[+mC]=Act5C-GAL4}25FO1/CyO, y[+];+* and Bloomington stock 2475, w[*]; *T(2;3)ap[Xa]*, *ap[Xa]*/*CyO*; *TM3*, *Sb[1]*.

Select against TM3, Sb[1] and any y[1] in subsequent generations to generate *w[*] ; {Act5C-GAL4}/ CyO ; {Ud}86/{Ud}86*

used to initiate population experiments. Full genotype is

*w[*];CyO/ P{w[+mC]=Act5C-GAL4}25FO1; M{3x-P3-RFP, {attR, w^[+mC]^ , , RpL14^[r]^, UAS-RpL14.dsRNA}}86Fb*

While all markers shown in the above figure need to be scored, elements targeted for the final stock are highlighted in color to make it more easy to follow.

The ‘wildtype’ stock used to initiate population experiments *w[*] ; {Act5C-GAL4}/ CyO : +/+* was generated at the same time but selecting against RFP in the final cross.

**Figure S6. Specificity of TaqMan probes for RpL14^[+]^ (FAM) and RpL14^[r]^ (VIC) transcripts.**


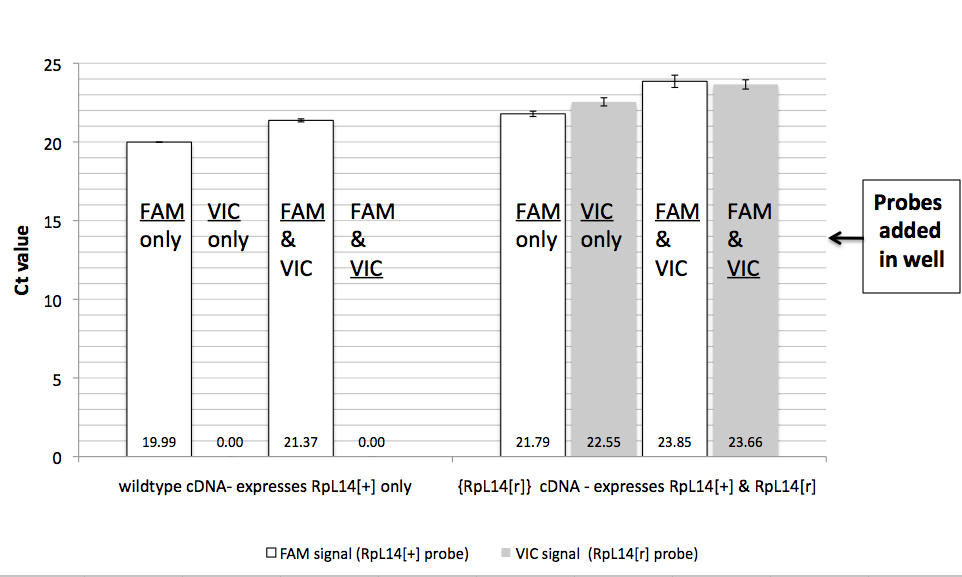


Probe specificity was assayed using cDNA from mixed sex pools of wildtype adults and from stocks that also express the rescue gene (but not the RNAi gene). Taqman PCR reactions were run with the FAM and VIC labeled probes added singly and jointly (see bar labels). Measured probe signal is represented as separate bars for each probe FAM (white) and VIC (grey), also indicated by the probe being underlined in the bar label. Error bars represent 1 standard error for three technical replicates. When both probes are present in a reaction with a wildtype cDNA sample template, signal is only observed from the FAM probe (which is designed to hybridize to the RpL14^[+]^ transcript). The VIC probe (designed to hybridize to the RpL14^[r]^ transcript) exhibits no detectable affinity for the RpL14^[+]^ transcript. There is a slight increase (~0.7) in Ct values in the FAM signal when both probes were present together, indicating some interaction between the probes. This small increase in signal in wells with both probes versus one probe was also apparent in assays with cDNA from individuals also expressing the rescue transcript (RpL14^[r]^ in addition to the wildtype transcript RpL14^[+]^). Despite the limited probe interaction, the relative amplification of FAM and VIC products were basically unchanged regardless of whether one or both probes are present in a well. This indicates that the VIC and the FAM probes are suitably specific to their intended targets.

**Figure S7. Genotypic levels of total *RpL14* mRNA expression without GAL4 expression in groups of whole individuals.**

Amount of *RpL14* mRNA in adult male genotypes and adult females in the absence of *Act5C-GAL4* expression, relative to three normalization genes. Legend as Figure 3, which shows the other relevant genotypes to the same scale. Height of bars indicate total amount of *RpL14* based on SYBR green-based quantitative reverse-transcription PCR. Each bar is split to represent the proportion of total *RpL14* expressed from the *RpL14^[r]^* gene in *{Ud}86* (white) and the endogenous *RpL14^[+]^*gene (grey), based on gene-specific TaqMan probes. Error bars represent 1 standard error for three biological replicates. The scale is identical to that used in Figure 3, where a total expression value of 1 corresponds to expression in wildtype males.

**Table S1. Data from multigenerational population experiments.**

The following gives the counts of RFP positive (RFP+) and RFP negative (RFP-), males (m) and females (f) each generation. Only generations 2-7, where heterozygotes could be present over the entire lifecycle, were used for plotting and fitness inference. At generation 2 line G was established from the females of line A after clearing them from A, line H was established from B, I from C, J from D, K from E, and L from F in the same manner. The estimated {*Ud*}*86* insert frequency is also given along with the total number of adults scored. Scoring was discontinued in replicates that had reached apparent loss or fixation for two generations.

| Replicate A |  |  |  |  |  |  |
| --- | --- | --- | --- | --- | --- | --- |
| Generation | RFP+ m | RFP+ f | RFP- m | RFP- f | {Ud86} frequency | number scored |
| 0 | 0 | 8 | 0 | 8 | 0.5 | 16 |
| 1 | 17 | 13 | 6 | 10 | 0.652174 | 46 |
| 2 | 44 | 29 | 18 | 5 | 0.510527 | 96 |
| 3 | 33 | 34 | 20 | 13 | 0.425544 | 100 |
| 4 | 16 | 32 | 28 | 25 | 0.275602 | 101 |
| 5 | 15 | 15 | 37 | 33 | 0.16334 | 100 |
| 6 | 3 | 6 | 50 | 43 | 0.045136 | 102 |
| 7 | 2 | 0 | 50 | 48 | 0.010051 | 100 |

| Replicate B |  |  |  |  |  |  |
| --- | --- | --- | --- | --- | --- | --- |
| Generation | RFP+ m | RFP+ f | RFP- m | RFP- f | {Ud86} frequency | number scored |
| 0 | 0 | 12 | 0 | 3 | 0.8 | 15 |
| 1 | 21 | 37 | 6 | 5 | 0.84058 | 69 |
| 2 | 41 | 45 | 9 | 5 | 0.625834 | 100 |
| 3 | 45 | 49 | 3 | 3 | 0.755051 | 100 |
| 4 | 58 | 48 | 1 | 0 | 0.903326 | 107 |
| 5 | 38 | 64 | 0 | 0 | 1 | 102 |
| 6 | 49 | 53 | 0 | 1 | 0.901467 | 103 |
| 7 | 47 | 54 | 0 | 0 | 1 | 101 |

| Replicate C |  |  |  |  |  |  |
| --- | --- | --- | --- | --- | --- | --- |
| Generation | RFP+ m | RFP+ f | RFP- m | RFP- f | {Ud86} frequency | number scored |
| 0 | 0 | 3 | 0 | 12 | 0.2 | 15 |
| 1 | 8 | 4 | 16 | 22 | 0.24 | 50 |
| 2 | 18 | 19 | 31 | 32 | 0.206275 | 100 |
| 3 | 8 | 3 | 39 | 51 | 0.056025 | 101 |
| 4 | 4 | 1 | 53 | 42 | 0.025321 | 100 |
| 5 | 3 | 0 | 44 | 53 | 0.015114 | 100 |
| 6 | 0 | 0 | 60 | 44 | 0 | 104 |
| 7 | 0 | 0 | 55 | 43 | 0 | 98 |

| Replicate D | |  |  |  |  |  |  |
| --- | --- | --- | --- | --- | --- | --- | --- |
| Generation | RFP+ m | | RFP+ f | RFP- m | RFP- f | {Ud86} frequency | number scored |
| 0 | 0 | | 8 | 0 | 8 | 0.5 | 16 |
| 1 | 6 | | 10 | 17 | 12 | 0.355556 | 45 |
| 2 | 30 | | 33 | 18 | 19 | 0.391724 | 100 |
| 3 | 20 | | 18 | 26 | 36 | 0.212599 | 100 |
| 4 | 19 | | 15 | 29 | 37 | 0.187596 | 100 |
| 5 | 1 | | 4 | 48 | 50 | 0.024574 | 103 |
| 6 | 1 | | 0 | 49 | 48 | 0.005115 | 98 |
| 7 | 0 | | 0 | 47 | 55 | 0 | 102 |

| Replicate E |  |  |  |  |  |  |
| --- | --- | --- | --- | --- | --- | --- |
| Generation | RFP+ m | RFP+ f | RFP- m | RFP- f | {Ud86} frequency | number scored |
| 0 | 0 | 12 | 0 | 3 | 0.8 | 15 |
| 1 | 6 | 11 | 2 | 0 | 0.894737 | 19 |
| 2 | 47 | 49 | 2 | 2 | 0.8 | 100 |
| 3 | 48 | 49 | 1 | 2 | 0.826795 | 100 |
| 4 | 48 | 52 | 0 | 0 | 1 | 100 |
| 5 | 45 | 44 | 0 | 0 | 1 | 89 |
| 6 |  |  |  |  |  |  |
| 7 |  |  |  |  |  |  |

| Replicate F |  |  |  |  |  |  |
| --- | --- | --- | --- | --- | --- | --- |
| Generation | RFP+ m | RFP+ f | RFP- m | RFP- f | {Ud86} frequency | number scored |
| 0 | 0 | 3 | 0 | 12 | 0.2 | 15 |
| 1 | 6 | 3 | 14 | 16 | 0.230769 | 39 |
| 2 | 14 | 12 | 42 | 33 | 0.138273 | 101 |
| 3 | 3 | 1 | 46 | 50 | 0.020204 | 100 |
| 4 | 0 | 0 | 42 | 58 | 0 | 100 |
| 5 | 0 | 0 | 48 | 52 | 0 | 100 |
| 6 |  |  |  |  |  |  |
| 7 |  |  |  |  |  |  |

| Replicate G | |  |  |  |  |  |  |
| --- | --- | --- | --- | --- | --- | --- | --- |
| Generation | RFP+ m | | RFP+ f | RFP- m | RFP- f | {Ud86} frequency | number scored |
| 0 |  | |  |  |  |  |  |
| 1 |  | |  |  |  |  |  |
| 2 | 44 | | 29 | 18 | 5 | 0.510527 | 96 |
| 3 | 32 | | 44 | 9 | 10 | 0.552786 | 95 |
| 4 | 38 | | 34 | 16 | 16 | 0.4453 | 104 |
| 5 | 43 | | 36 | 16 | 9 | 0.50971 | 104 |
| 6 | 17 | | 50 | 4 | 29 | 0.425544 | 100 |
| 7 | 16 | | 17 | 32 | 33 | 0.185589 | 98 |

| Replicate H | |  |  |  |  |  |  |
| --- | --- | --- | --- | --- | --- | --- | --- |
| Generation | RFP+ m | | RFP+ f | RFP- m | RFP- f | {Ud86} frequency | number scored |
| 0 |  | |  |  |  |  |  |
| 1 |  | |  |  |  |  |  |
| 2 | 41 | | 45 | 9 | 5 | 0.625834 | 100 |
| 3 | 39 | | 59 | 2 | 0 | 0.858579 | 100 |
| 4 | 50 | | 53 | 0 | 0 | 1 | 103 |
| 5 | 58 | | 40 | 0 | 0 | 1 | 98 |
| 6 |  | |  |  |  |  |  |
| 7 |  | |  |  |  |  |  |

| Replicate I |  |  |  |  |  |  |
| --- | --- | --- | --- | --- | --- | --- |
| Generation | RFP+ m | RFP+ f | RFP- m | RFP- f | {Ud86} frequency | number scored |
| 0 |  |  |  |  |  |  |
| 1 |  |  |  |  |  |  |
| 2 | 18 | 19 | 31 | 32 | 0.206275 | 100 |
| 3 | 4 | 4 | 57 | 35 | 0.040834 | 100 |
| 4 | 2 | 0 | 41 | 60 | 0.009756 | 103 |
| 5 | 3 | 0 | 44 | 54 | 0.014963 | 101 |
| 6 | 0 | 0 | 45 | 52 | 0 | 97 |
| 7 | 0 | 0 | 48 | 53 | 0 | 101 |

| Replicate J |  |  |  |  |  |  |
| --- | --- | --- | --- | --- | --- | --- |
| Generation | RFP+ m | RFP+ f | RFP- m | RFP- f | {Ud86} frequency | number scored |
| 0 |  |  |  |  |  |  |
| 1 |  |  |  |  |  |  |
| 2 | 30 | 33 | 18 | 19 | 0.391724 | 100 |
| 3 | 14 | 30 | 27 | 30 | 0.248763 | 101 |
| 4 | 14 | 24 | 26 | 36 | 0.212599 | 100 |
| 5 | 15 | 13 | 40 | 35 | 0.14668 | 103 |
| 6 | 13 | 7 | 46 | 36 | 0.103383 | 102 |
| 7 | 4 | 5 | 49 | 50 | 0.042573 | 108 |

| Replicate K |  |  |  |  |  |  |
| --- | --- | --- | --- | --- | --- | --- |
| Generation | RFP+ m | RFP+ f | RFP- m | RFP- f | {Ud86} frequency | number scored |
| 0 |  |  |  |  |  |  |
| 1 |  |  |  |  |  |  |
| 2 | 47 | 49 | 2 | 2 | 0.8 | 100 |
| 3 | 37 | 61 | 1 | 0 | 0.899496 | 99 |
| 4 | 48 | 58 | 1 | 0 | 0.903326 | 107 |
| 5 | 38 | 62 | 0 | 0 | 1 | 100 |
| 6 | 50 | 50 | 0 | 0 | 1 | 100 |
| 7 |  |  |  |  |  |  |

| Replicate L |  |  |  |  |  |  |
| --- | --- | --- | --- | --- | --- | --- |
| Generation | RFP+ m | RFP+ f | RFP- m | RFP- f | {Ud86} frequency | number scored |
| 0 |  |  |  |  |  |  |
| 1 |  |  |  |  |  |  |
| 2 | 14 | 12 | 42 | 33 | 0.138273 | 101 |
| 3 | 10 | 0 | 54 | 36 | 0.051317 | 100 |
| 4 | 0 | 1 | 46 | 53 | 0.005013 | 100 |
| 5 | 0 | 0 | 38 | 62 | 0 | 100 |
| 6 | 0 | 0 | 44 | 56 | 0 | 100 |
| 7 |  |  |  |  |  |  |

**Table S2. Data from the development time and genotype viability experiments.**

The counts of offspring from the various types of crosses presented in Figure S5 are given below. The nine offspring with observed genotypes that were not possible in the cross setup are underlined and in bold. These were used to estimate the genotyping error rate and were excluded from all other calculations. The female parent is listed first. Male offspring are indicated by “m” and female offspring by “f.” Totals of survivors for each genotype are presented for both sexes at the end of the table.

{Ud}86/+ × {Ud}86/+

| Day | 9 | 10 | 11 | 12 | 13 | 14 | 15 | 16 | 17 | 18 | 19 | 20 | 21 |
| --- | --- | --- | --- | --- | --- | --- | --- | --- | --- | --- | --- | --- | --- |
| +/+ m | 0 | 50 | 103 | 68 | 29 | 24 | 10 | 9 | 0 | 3 | 1 | 1 | 0 |
| {Ud}86/+ m | 0 | 8 | 122 | 107 | 84 | 52 | 46 | 24 | 6 | 5 | 2 | 0 | 0 |
| {Ud}86/{Ud}86 m | 0 | 39 | 94 | 63 | 22 | 26 | 14 | 12 | 3 | 0 | 0 | 2 | 0 |
| +/+ f | 2 | 82 | 81 | 52 | 36 | 25 | 14 | 9 | 4 | 4 | 1 | 0 | 0 |
| {Ud}86/+ f | 0 | 28 | 144 | 124 | 84 | 78 | 47 | 16 | 8 | 1 | 3 | 1 | 0 |
| {Ud}86/{Ud}86 f | 0 | 85 | 103 | 68 | 37 | 21 | 16 | 4 | 2 | 3 | 0 | 0 | 0 |

+/+ × {Ud}86/+

| Day | 9 | 10 | 11 | 12 | 13 | 14 | 15 | 16 | 17 | 18 | 19 | 20 | 21 |
| --- | --- | --- | --- | --- | --- | --- | --- | --- | --- | --- | --- | --- | --- |
| +/+ m | 0 | 3 | 60 | 105 | 38 | 17 | 4 | 2 | 1 | 3 | 0 | 0 | 0 |
| {Ud}86/+ m | 0 | 0 | 23 | 49 | 62 | 27 | 12 | 3 | 2 | 2 | 1 | 0 | 0 |
| {Ud}86/{Ud}86 m | 0 | 0 | 0 | 0 | 0 | 0 | 0 | 0 | 0 | 0 | 0 | 0 | 0 |
| +/+ f | 0 | 11 | 115 | 98 | 38 | 6 | 2 | 3 | 2 | 1 | 0 | 0 | 0 |
| {Ud}86/+ f | 0 | 1 | 22 | 106 | 60 | 26 | 16 | 5 | 1 | 2 | 0 | 1 | 0 |
| {Ud}86/{Ud}86 f | 0 | 0 | 0 | 0 | 0 | 0 | 0 | 0 | 0 | 0 | 0 | 0 | 0 |

{Ud}86/{Ud}86 × {Ud}86/+

| Day | 9 | 10 | 11 | 12 | 13 | 14 | 15 | 16 | 17 | 18 | 19 | 20 | 21 |
| --- | --- | --- | --- | --- | --- | --- | --- | --- | --- | --- | --- | --- | --- |
| +/+ m | 0 | 0 | 0 | 0 | 0 | 0 | 0 | 0 | 0 | 0 | 0 | 0 | 0 |
| {Ud}86/+ m | 0 | 0 | 18 | 43 | 43 | 29 | 12 | 2 | 0 | 0 | 0 | 0 | 0 |
| {Ud}86/{Ud}86 m | 0 | 10 | 65 | 72 | 25 | 11 | 6 | 0 | 0 | 0 | 0 | 0 | 0 |
| +/+ f | 0 | 0 | 0 | 0 | 0 | 0 | 0 | 0 | 0 | 0 | 0 | 0 | 0 |
| {Ud}86/+ f | 0 | 0 | 12 | 75 | 55 | 34 | 9 | 1 | 1 | 0 | 0 | 1 | 0 |
| {Ud}86/{Ud}86 f | 0 | 11 | 96 | 91 | 26 | 7 | 7 | 0 | 1 | 0 | 0 | 0 | 0 |

{Ud}86/+ × +/+

| Day | 9 | 10 | 11 | 12 | 13 | 14 | 15 | 16 | 17 | 18 | 19 | 20 | 21 |
| --- | --- | --- | --- | --- | --- | --- | --- | --- | --- | --- | --- | --- | --- |
| +/+ m | 0 | 0 | 21 | 49 | 13 | 1 | 1 | 0 | 0 | 1 | 0 | 0 | 0 |
| {Ud}86/+ m | 0 | 0 | 2 | 22 | 33 | 14 | 2 | 1 | 0 | 0 | 0 | 1 | 0 |
| {Ud}86/{Ud}86 m | 0 | 0 | 0 | **1** | **3** | 0 | 0 | 0 | 0 | 0 | 0 | 0 | 0 |
| +/+ f | 0 | 0 | 52 | 52 | 9 | 0 | 2 | 0 | 0 | 0 | 1 | 0 | 0 |
| {Ud}86/+ f | 0 | 0 | 4 | 42 | 26 | 11 | 3 | 1 | 0 | 0 | 0 | 0 | 0 |
| {Ud}86/{Ud}86 f | 0 | 0 | 0 | 0 | **2** | 0 | 0 | 0 | 0 | 0 | 0 | 0 | 0 |

{Ud}86/+ × {Ud}86/{Ud}86

| Day | 9 | 10 | 11 | 12 | 13 | 14 | 15 | 16 | 17 | 18 | 19 | 20 | 21 |
| --- | --- | --- | --- | --- | --- | --- | --- | --- | --- | --- | --- | --- | --- |
| +/+ m | 0 | 0 | 0 | 0 | 0 | 0 | 0 | 0 | 0 | 0 | 0 | 0 | 0 |
| {Ud}86/+ m | 0 | 0 | 11 | 24 | 11 | 5 | 3 | 1 | 1 | 0 | 0 | 0 | 0 |
| {Ud}86/{Ud}86 m | 0 | 3 | 43 | 31 | 6 | 8 | 4 | 2 | 1 | 0 | 0 | 0 | 0 |
| +/+ f | 0 | 0 | 0 | 0 | 0 | 0 | **3** | 0 | 0 | 0 | 0 | 0 | 0 |
| {Ud}86/+ f | 0 | 0 | 16 | 17 | 16 | 5 | 7 | 1 | 0 | 0 | 0 | 0 | 0 |
| {Ud}86/{Ud}86 f | 0 | 11 | 64 | 26 | 19 | 9 | 3 | 2 | 1 | 0 | 0 | 0 | 0 |

Totals

|  | +/+ | +/+ | {Ud}86/+ | {Ud}86/+ | {Ud}86/{Ud}86 | {Ud}86/{Ud}86 |
| --- | --- | --- | --- | --- | --- | --- |
| Parental cross | m | f | m | f | m | f |
| {Ud}86/+ × {Ud}86/+ | 298 | 310 | 456 | 534 | 275 | 339 |
| +/+ × {Ud}86/+ | 233 | 276 | 181 | 240 | 0 | 0 |
| {Ud}86/{Ud}86 × {Ud}86/+ | 0 | 0 | 147 | 188 | 189 | 239 |
| {Ud}86/+ × +/+ | 86 | 116 | 75 | 87 | **4** | **2** |
| all crosses | 617 | 702 | 859 | 1049 | 468 | 580 |

**Table S3. Dry Weight Data**

Dry weights in grams for batches of 10 flies weighed at a time for each sex, replicate and genotype.

| Females |  |  |  |
| --- | --- | --- | --- |
| Genotype | {Ud}86/{Ud}86 | {Ud}86/+ | +/+ |
| Replicate 1 | 0.0031 | 0.0032 | 0.0031 |
| Replicate 2 | 0.003 | 0.0031 | 0.0032 |
| Replicate 3 | 0.0037 | 0.003 | 0.0032 |
| Replicate 4 | 0.0033 | 0.0038 | 0.0031 |
| Replicate 5 | 0.0033 | 0.0034 | 0.0026 |
| Mean | 0.00328 | 0.0033 | 0.00304 |

| Males |  |  |  |
| --- | --- | --- | --- |
| Genotype | {Ud}86/{Ud}86 | {Ud}86/+ | +/+ |
| Replicate 1 | 0.002 | 0.0022 | 0.0022 |
| Replicate 2 | 0.0022 | 0.0023 | 0.0023 |
| Replicate 3 | 0.0024 | 0.0022 | 0.0025 |
| Replicate 4 | 0.0026 | 0.0028 | 0.0022 |
| Replicate 5 | 0.0024 | 0.0026 | 0.0022 |
| Mean | 0.00232 | 0.00242 | 0.00228 |

**Text S1. Supplementary equations.**

**Supplementary equations**

(Numbers in square brackets refer to the references given in the main text and numbers in parentheses refer to numbered equations)

Geographic stability and dynamics of underdominant population transformation

Here, we give some insight into the quantitative analysis of geographic stability properties of underdominant population transformation with *{Ud}* constructs. Optimal release strategies have been assessed [1,2] for a simple but insightful meaningful geographic pattern, but also need to be addressed in future work. The mathematical methods presented here serve as a quantitative justification for the geographic stability properties discussed in the main text.

We assume that transformation of a target population has already been achieved and are interested in the systems potential of further spread of *{Ud}* compared to the possibility of complete loss of *{Ud}*. The mathematical evolutionary model is based on genotypic fitness parameters, migration rates between neighboring populations, and population size, which determine the extinction processe’s intrinsic time scale.

In a single locus two allele model the genotypic fitness values that determine the population dynamics of our system are $w_{+/+}$, $w_{+/\{Ud\}86}$, and $w_{\{Ud\}86\{Ud\}86}$. For our analytical and simulation results we use the MLE fitness estimates of the transgenic genotypes, figure 2 (C), $w_{+/+}=1$, $w_{+/\{Ud\}86}=0.22$, and $w_{\{Ud\}86\{Ud\}86}=0.71$. Under random mating (random union of gametes) we can assume that diploid individuals pass through Hardy-Weinberg expectations before selection, and thus describe the population system in terms of single alleles [3,4]. The frequency threshold to transform an isolated target population neglecting migration is

1. $\breve{\rho}=\frac{w_{+/+}-w_{+/\{Ud\}}}{w_{+/+}+w_{\{Ud\}/\{Ud\}}-2w_{+/\{Ud\}}}$,

which determines an unstable equilibrium of the evolutionary dynamics. Given that the target population has been transformed, we can estimate the two further threshold allele frequencies, respecting migration of gametes prior to mating. First, in a neighboring population we can calculate an approximation for the threshold to transform the neighboring population under the assumption that the target population is unaffected. Second, we can approximate the threshold for a transformation of the target population back to wildtype due to migrants from neighboring wild populations.

For our migration-selection dynamics, the expected allelic fitness values are affected by the migration rate per generation (or any other unit of time), but the genotypic fitness values remain constant. The rate of immigrants into a given population prior to mating is given by $m$. The expected allelic fitness values depend on the allele frequencies, and thus on migration rate. Under the Hardy-Weinberg assumption all fitness values can be expressed in terms of the frequency of *{Ud}*:$p_{T}$in the target population, and $p_{N}$in a neighboring population. For a wildtype allele $+$, we have

$$f_{+}=(1-p_{T})w_{+/+}+p_{T}w_{+/\{Ud\}}+m\left(p_{T}-p_{N}\right)\left(w_{+/+}-w_{+/\{Ud\}}\right)$$

Likewise, for the underdominant construct allele *{Ud}*, we have

$$f_{\{Ud\}}=(1-p_{T})w_{+/\{Ud\}}+p_{T}w_{\{Ud\}/\{Ud\}}-m\left(p_{T}-p_{N}\right)\left(w_{\{Ud\}/\{Ud\}}-w_{+/\{Ud\}}\right)$$

In the second terms of Equations (2), and (3) we see an important asymmetry in the effect of migration on the average fitness values of the two alleles, which eventually leads to the asymmetry described in Figure 4. The average population fitness in the target population is

$$F_{T}=\left(1-p_{T}\right)f_{+}+p_{T}f_{\{Ud\}}-m\left(p_{T}-p_{N}\right)\left(f_{\{Ud\}}-f_{+}\right)$$

With the fitness functions (2), (3), and (4) we can describe the temporal change of the frequencies of allele *{Ud}* in the target population,$\Delta p_{T}$, and in the neighboring population,$\Delta p_{N}$, per time step$\Delta t$(typically one generation) [2–4].

$$\Delta p_{T}(p_{T},p_{N})=\Delta tp_{T}\left(f_{\{Ud\}}-F_{T}\right)-m\Delta t\left(p_{T}-p_{N}\right)f_{\{Ud\}}$$

The equation for $\Delta p_{N}$follows by exchanging $p_{N}$and $p_{T}$, $\Delta p_{N}(p_{T},p_{N})=\Delta p_{T}(p_{N},p_{T})$.

We use the two equations for the evolutionary dynamics of the frequency of *{Ud}* in target and neighboring populations to give quantitative estimates for the bi-stable properties and geographic stability in infinitely large populations, Figures 2 and 4. The calculation of the thresholds used in Figure 4 then follows from finding the roots of $\Delta p_{T}(\rho_{T},0)=0$, and $\Delta p_{N}(\rho_{N},1)=0$: For back-transformation of the target population due to immigration from neighboring populations $p_{T}$ has to be below $\rho_{N}$, *i.e.*, one has to increase wildtype from $0$ to $1-\rho_{T}$frequency. In wild populations, $\rho_{N}$ is the threshold for transforming a single neighboring population due to emigration from the transformed target. In the following we set the constant fitness of wildtype homozygotes to one, all other fitness values can be given in relative terms, $w_{+/+}=1$. With this the thresholds we are interested in can be found to have the following form

$$\rho_{T}=\frac{A_{T}+\sqrt{B_{T}}}{2\left(1-m\right)\left(1+w_{\{Ud\}/Ud\}}-2w_{+/Ud\}}\right)}$$

$$\rho_{N}=\frac{A_{N}-\sqrt{B_{N}}}{2\left(1-m\right)\left(1+w_{\{Ud\}/Ud\}}-2w_{+/Ud\}}\right)}$$

with

$$A_{T}=2\left(1-w_{\{Ud\}/\{Ud\}}\right)+\left(1-m\right)\left(w_{\{Ud\}/\{Ud\}}-w_{+/\{Ud\}}\right)$$

$$A_{N}=1-w_{+/\{Ud\}}-m\left(1-2w_{\{Ud\}/\{Ud\}}-3w_{+/\{Ud\}}\right)$$

and

$$B_{T}=\left(1-m\right)^{2}\left(w_{\{Ud\}/\{Ud\}}-w_{+/\{Ud\}}\right)^{2}-4m\left(w_{\{Ud\}/\{Ud\}}-\left(w_{+/\{Ud\}}\right)^{2}\right)$$

$$B_{N}=\left(1-m\right)^{2}\left(1-w_{+/\{Ud\}}\right)^{2}-4m\left(w_{\{Ud\}/\{Ud\}}-\left(w_{+/\{Ud\}}\right)^{2}\right)$$

There can only be meaningful results as long as $B_{T}$, and$B_{N}$ are nonnegative, which holds for migration below critical thresholds. As migration goes to zero, both thresholds converge to the well-known threshold in an isolated population, Equation (1). It is also easy to see that that underdominance means $\rho_{N}\leq\rho_{T}$for migration below critical bounds. This asymmetry is responsible for the self-limiting property: loss is more likely than spread [2].

Equations (2), (3), and (4) can also be used to mathematically model stochastic dynamics in finite populations, either using a Moran or Wright-Fisher approach [2,3]. Such simulations also reveal that only (effective) population sizes of a magnitude below 100 females have a realistic chance of undesired spread to a neighboring population (assuming no mating discrimination and migration prior to mating).

**References cited in supplementary materials (all are also in main text bibliography)**

1. Marshall JM, Hay BA (2012) Confinement of gene drive systems to local populations : A comparative analysis. J Theor Biol 294: 153–171. doi:10.1016/j.jtbi.2011.10.032.

2. Altrock PM, Traulsen A, Reed FA (2011) Stability Properties of Underdominance in Finite Subdivided Populations. PLoS Comput Biol 7: e1002260. doi:10.1371/journal.pcbi.1002260.

3. Altrock PM, Traulsen A, Reeves RG, Reed FA (2010) Using underdominance to bi-stably transform local populations. J Theor Biol 267: 62–75. doi:10.1016/j.jtbi.2010.08.004.

4. Hartl DL, Clark AG (1997) Principles of Population Genetics. Sinauer AD, editor Sinauer Associates.
